# Factors affecting RFID adoption in the agricultural product distribution industry: empirical evidence from China

**DOI:** 10.1186/s40064-016-3708-x

**Published:** 2016-11-28

**Authors:** Ping Shi, Bo Yan

**Affiliations:** 1 School of Management, Guangdong University of Technology, Guangzhou, 510520 China; 2 School of Economics and Commerce, South China University of Technology, Guangzhou, 510006 China

**Keywords:** Technology adoption, Agricultural product distribution industry, RFID, Structural equation modeling (SEM), Technology–organization–environment (TOE)

## Abstract

We conducted an exploratory investigation of factors influencing the adoption of radio frequency identification (RFID) methods in the agricultural product distribution industry. Through a literature review and field research, and based on the technology–organization–environment (TOE) theoretical framework, this paper analyzes factors influencing RFID adoption in the agricultural product distribution industry in reference to three contexts: technological, organizational, and environmental contexts. An empirical analysis of the TOE framework was conducted by applying structural equation modeling based on actual data from a questionnaire survey on the agricultural product distribution industry in China. The results show that employee resistance and uncertainty are not supported by the model. Technological compatibility, perceived effectiveness, organizational size, upper management support, trust between enterprises, technical knowledge, competitive pressure and support from the Chinese government, which are supported by the model, have significantly positive effects on RFID adoption. Meanwhile, organizational size has the strongest positive effect, while competitive pressure levels have the smallest effect. Technological complexities and costs have significantly negative effects on RFID adoption, with cost being the most significantly negative influencing factor. These research findings will afford enterprises in the agricultural products supply chain with a stronger understanding of the factors that influence RFID adoption in the agricultural product distribution industry. In addition, these findings will help enterprises remain aware of how these factors affect RFID adoption and will thus help enterprises make more accurate and rational decisions by promoting RFID application in the agricultural product distribution industry.

## Background

The safety of agricultural products has become a central societal issue, as safety not only affects consumer health but can also devastate agricultural produce enterprises and the entire agricultural supply chain, seriously affecting the sustainable development of agricultural product industries and societal stability. As an important aspect of food safety management, the traceability of agricultural products plays a key role. China in particular is becoming the world’s largest food producer and consumer. Related policies that involve setting up uniform tracing information platforms and enacting standards are promoted widely. However, owing to the lengthy and dynamic nature of supply chains, traditionally wired sensor technologies can barely address agricultural product monitoring needs. With the rapid development of the Internet of things (IOT) industry, radio frequency identification (RFID) has been applied extensively around the world, and some developed countries have achieved favorable results by using RFID to manage agricultural supply chain safety, in turn attracting interest from academics. Most studies on agricultural products based on RFID technologies have focused on their applications, while the introduction of new technologies typically requires rigorous and scientific analysis to satisfy industry development requirements. Factors influencing RFID adoption have not yet been studied. By studying these issues, China can begin to promote agricultural information management systems.

## Literature review and our contributions

### Literature review

Many scholars have studied the applications of RFID for agricultural product management by exploring applications of RFID for agricultural products (Sahin et al. 2002; Regattieri et al. 2007; Amador et al. 2009; Abad et al. 2009), the construction of agricultural product information systems (Gras 2006; Bernardi et al. 2007; Liu and Tang 2010), and the quality management of agricultural products based on RFID (Bernardi et al. 2007; Xie et al. 2007; Jedermanna et al. 2009). While it is worthwhile to study applications of RFID in the agricultural product distribution industry, few studies have examined factors that influence RFID adoption in agricultural product supply chains. In practice, RFID technologies have been implemented widely in some developed countries through governmental and business projects (e.g., Wal-Mart and DHL). Although the IOT industry is growing in China, related successful projects have been rare, with most still occupying the experimental stage. China has lagged behind other developed counties in terms of technological, standard, supply chain and application development. Most related studies have attributed this to lower labor costs in China, to the higher costs of RFID adoption and to undeveloped standards and regulations (Zhang 2011). The introduction of RFID into the agricultural product distribution industry thus serves as an important premise for analyzing factors influencing RFID adoption.

Information technology adoption theory constitutes an emerging facet of information systems research. It examines the behavioral characteristics of organizations and individuals as they adopt and accept information technologies based on principles of social psychology and behavioral science to determine ways in which users accept and continue to use information technologies (Li 2006). The theory plays an important role in studying the introduction of RFID technologies into the management of agricultural products. Fichman (1992) summarized numerous factors influencing information technology adoption and classified information technology adoption theories into two categories: theories focused on individual-level adoption behaviors and those focused on organizational-level adoption behaviors. There are relatively few organization-level information technology adoption behavior theories (Li 2011). Some classic theories include Innovation Diffusion Theory (IDT), the six-stage model, and the TOE analysis framework. Tornatzky and Fleischer (1990) criticized IDT and maintained that classical innovation diffusion theory is critical of the notion that factors affecting information technology adoption include not only technology elements (T) but also characteristic elements of organizations (O) and environmental elements (E). After the technology–organization–environment (TOE) framework was first proposed, many scholars began to study TOE theory and related influencing factors due to its applicability. TOE theory considers technological issues in terms of technical compatibilities, complexities, observability levels, etc. as well as organizational factors such as the size of an organization, high-level support received, organizational cultures, etc. and environmental factors such as external competitive pressures and government policy support. Chau and Tam (1997) used the TOE framework to analyze factors that affect open-system adoption. Kuan and Chau (2001) proposed a perception-based small business EDI adoption model tested against data collected from 575 small firms in Hong Kong based on the TOE theoretical framework. Grandon and Pearson (2004) examined determinant factors of strategic value and electronic commerce adoption as perceived by upper managers in small- and medium-sized enterprises in the Midwest region of the US. Zhang and Kang (2008) analyzed influencing factors and countermeasures of logistics information network technologies. Li (2011) designed a process model for analyzing factors shaping RFID adoption in the automobile manufacturing industry and applied it through a case study on China’s automobile manufacturing industry.

Therefore, RFID technology application in the agricultural product distribution industry is bound to be affected by various factors. Identifying these factors will play a key role in RFID technology adoption. Processes and node enterprises in agricultural product supply chains constitute an organizational system. It is necessary to analyze factors influencing RFID adoption through an examination of entire organizations. We thus use organizational level adoption behavior theory to conduct this study. TOE theory takes into account technical, organizational and environmental factors, thus forming a more comprehensive framework.

### Our paper and contributions

From our study and summary of relevant literature, we draw the following conclusions:

1. Research on factors influencing RFID adoption in organizations is still relatively new, and with respect to research focused on China, only Li (2011) designed a model for analyzing factors influencing RFID adoption in China’s automobile manufacturing industry.
2. For research methods, researchers initially used qualitative analysis methods such as literature reviews, case studies, and interviews with experts. More recently, researchers have conducted quantitative analyses such as questionnaires and statistical analyses.
3. In terms of research models, most related literature has employed Rogers’ diffusion of innovation theory (Rogers 1983). Other researchers prefer the TOE framework proposed by Tornatzky and Fleischer (1990).

Technological, organizational, and environmental contexts form the basis of our comprehensive research framework, and factors shaping RFID adoption within each category are highlighted. From our literature review, factors influencing RFID adoption in the agricultural product distribution industry are preliminarily summarized in Table 1. While Chinese governmental support is rarely referenced in the extant literature, after contacting experts of this area, we found that the Chinese government plays an important role in the adoption of new information technologies. We thus take support from the Chinese government into consideration.

**Table 1:** The frequency of references to factors influencing RFID adoption in the related literature

Categories	Influencing factors	Times
Technological context	Technological complexity	5
	Technological compatibility	3
	Perceived effectiveness	16
	Cost	6
Organizational context	Organizational size	3
	Upper management support	6
	Trust between enterprises	2
	Technical knowledge	4
	Employee resistance	2
Environmental context	Competitive pressure	10
	Uncertainty	4
	Chinese government support	1

In sum, we conduct an exploratory investigation of factors that influence RFID adoption in the agricultural product distribution industry. Using the TOE theoretical framework, we analyze factors that influencing RFID adoption in the agricultural product distribution industry based on the following three contexts: technological, organizational, and environmental contexts. We conduct an empirical analysis of the TOE framework by applying structural equation modeling (SEM) based on actual data drawn from a questionnaire survey on the agricultural product distribution industry in China.

## Hypotheses and the research model

### Technological context

1. Technological complexity

The more complex a form of technology is, the less possible it is for it to be successfully applied. When a form of technology is very difficult for an organization to apply, upper management teams determine to either abandon it or to introduce it later. Thus, we initially hypothesized that RFID complexity negatively affects adoption. Therefore, we propose the following:

#### **Hypothesis 1**

Technological complexity has negative effects on RFID adoption.

(2) Technological compatibility

We define technological compatibility here as the degree to which RFID corresponds with an organization’s business processes, IT infrastructure, distribution channels, corporate culture, and value system. Generally, it is easier for an organization to employ a form of information technology when it offers a higher degree of technological compatibility. Hence, the following hypothesis is proposed:

#### **Hypothesis 2**

Technological compatibility has a positive effect on RFID adoption.

(3) Perceived effectiveness

The presence of perceived effectiveness enables a higher degree of supply chain visualization, saves time costs, reduces human resource costs, improves business efficiency levels, etc. Therefore, we propose that:

#### **Hypothesis 3**

Perceived effectiveness has a positive effect on RFID adoption.

(4) Cost

Tornatzky and Klein (1982) showed that costs inhibit the adoption of new technologies. In this paper, costs range from hardware facility costs (including RFID/EPC tags, readers, sensors, middleware and servers) to costs of system implementation, integration, operation, and maintenance. Hence, the following hypothesis is proposed:

#### **Hypothesis 4**

Costs have a negative effect on RFID adoption.

### Organizational context

1. Organizational size

Large-scale enterprises have access to more resources for testing out new forms of technology and can then determine whether to adopt them or not. Meanwhile, they are more likely to achieve economies of scale and to reduce risks associated with new technology adoption. They are also better equipped to persuade supply chain partners to adopt new technologies. Hence, the following hypothesis is proposed:

#### **Hypothesis 5**

Organizational size has a positive effect on RFID adoption.

(2) Upper management support

When a new form of technology is adopted by an organization, this is bound to affect all aspects of the organization, altering business processes and organizational structures, which are all uncertain factors that shape new technology adoption. When faced with such uncertainties, decision makers tend to initially exercise caution. Despite the benefits of RFID adoption, as such adoption can change existing business processes and requires financial support from enterprises, upper management support is known to be critical to the adoption of new technologies in an organization. We thus propose the following:

#### **Hypothesis 6**

Upper management attitudes towards RFID positively affect RFID adoption.

(3) Trust between enterprises

When strong cooperation and trust is not present, freeriding is likely to occur with RFID adoption, in which case upstream suppliers bear most of the incurred costs (Geng 2005). Thus, we propose the following:

#### **Hypothesis 7**

Trust between enterprises has a positive effect on RFID adoption.

(4) Technical knowledge

Technical knowledge refers to professional IT knowledge owned by enterprises themselves. When they have grasped relevant knowledge and skills pertaining to a new form of technology, companies can effectively assess factors that influence the adoption of this new technology, including advantages, disadvantages, costs, etc. Therefore, the following hypothesis is proposed:

#### **Hypothesis 8**

Technical knowledge has a positive effect on RFID adoption.

(5) Employee resistance

When a new form of technology is adopted, some employees may think that they do not have the necessary qualifications or skills to operate this new form of technology. Meanwhile, as the introduction of new technologies increases operation efficiency levels while decreasing labor force requirements, employees will worry about losing their jobs and will therefore exhibit resistance to the adoption of new technologies. Hence, the following hypothesis is proposed:

#### **Hypothesis 9**

Employee resistance has a negative effect on RFID adoption.

### Environmental context

1. Competitive pressure

Premkumar and Ramamurthy (1995) discovered that due to internal pressures and a desire to gain a competitive advantage, enterprises must adopt new technologies. It is likely that they may also face not only pressures resulting from technological innovations generated by upstream and downstream partners in the supply chain and by competitors but also pressures resulting from new developments in business models and industry standards. Therefore, we propose the following:

#### **Hypothesis 10**

Competitive pressure has a positive effect on RFID adoption.

(2) Uncertainty

A lack of information and technical knowledge or being unable to forecast development patterns leads to uncertainty. It is quite typical for an enterprise to be unable to determine the requirements of their products or the number of loyal customers in the market. Therefore, the following hypothesis is proposed:

#### **Hypothesis 11**

Uncertainty has a negative effect on RFID adoption.

(3) Chinese government support

Policies and laws proposed by the Chinese government and financial support given will play an important role in promoting the adoption of new technologies. In China, many companies are government-oriented, and when a new form of technology is supported by the Chinese government, it will be widely adopted in the given industry quickly and easily. Hence, the following hypothesis is proposed:

#### **Hypothesis 12**

Support from the Chinese government has a positive effect on RFID adoption.

**Fig. 1:**
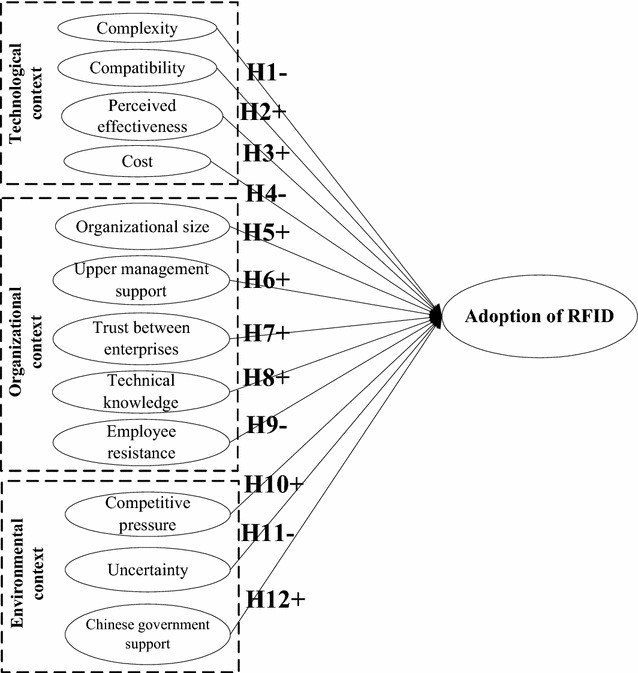
The research model

The research model developed through this study is illustrated in Fig. 1. “+” denotes that a factor has a positive effect on RFID adoption, whereas “−” denotes that a factor has a negative effect.

## Research methodology

### Samples and data collection

Due to the limited amount of time the managerial respondents could offer, a mail survey approach was used to allow respondents to complete the surveys at their convenience. For reliability and operability purposes, we recruited staff members who are aware of supply chains, RFID and information technology observations. Upper-level managers, middle managers, and professional staff from agricultural product distribution enterprises in Foshan and Guangzhou and Wal-Mart’s suppliers of agricultural products in Shenzhen were studied, as they are familiar with the requirements of running of a company and have a general understanding of new technologies such as RFID. Of these, 34 companies qualified and agreed to participate in the mailed survey. A contact person from each company was selected to distribute the questionnaire to relevant staff members. Concerning of the professionalism of our questionnaire, we actively communicated with respondents to ensure the authenticity and reliability of our results, as errors can emerge when respondents do not understand questions fully or when they ascribe too much subjective meaning to each question.

We studied 34 companies in total, and each company was sent 3–5 sections of the questionnaire unequally. In total, 150 survey packages were sent out. Each survey package included a cover letter, questionnaire, and stamped return envelope. In total, 92 completed questionnaires were returned. However, nine responses were discarded due to incomplete data. We received 83 valid responses, and the effective response rate was measured at 55.3%. Demographic features of the respondents surveyed are shown in Tables 2 and 3. The respondents were upper-level managers (8.7%), middle managers (21.7%), or professional staff members (69.6%). Regarding the size of each organization in terms of its number of employees, 2.9% of the responses were classified as responses from large firms (more than 1000 employees), 5.9% were classified as responses from medium-sized firms (501–999 employees), 26.5% were classified as responses from small firms (101–499 employees), and the remaining 64.7% were classified as responses from firms with less than 100 employees.

**Table 2:** Descriptive statistics on the respondent positions

Categories	Frequency	Percentage
Upper-level managers	8	8.7
Middle managers	20	21.7
Professional staffs	64	69.6

**Table 3:** Descriptive statistics on the respondent organization sizes

Categories	Frequency	Percentage
Large enterprises	1	2.9
Medium-sized enterprises	2	5.9
Small enterprises	9	26.5
Micro enterprises	22	64.7

### Measurement development

Measurement items used to operationalize the constructs were adapted from relevant previous studies. All scale items were rephrased to relate specifically to RFID and to the agricultural product supply chain. To facilitate our data analysis, each measurement item was measured on a 7-point Likert scale ranging from “strongly disagree” (=1) to “strongly agree” (=7). Based on the previous context, definitions and analyses of each measurement item are shown in Tables 4, 5, and 6.

**Table 4:** Measurement of technological contexts

Variable	Statement of measurement	References
Technological complexity	T11: RFID system operation is complex	Brown and Bakhru (2007) and Riggins and Slaughter (2006)
	T12: RFID system operation is inconvenient	
	T13: RFID system operation requires ample experience	
Technological compatibility	T21: RFID technologies are compatible with business processes	Rogers (1983) and Li (2011)
	T22: RFID technologies are compatible to other information systems (e.g., ERP, MIS and WMS)	
	T23: RFID technologies complement knowledge held by agricultural product distribution enterprise employees	
Perceived effectiveness	T31: RFID technologies make agricultural product supply chains more transparent and improve visualization capacities	Kuan and Chau (2001), Brown and Russel (2007) and Seymour et al. (2010)
	T32: RFID technologies reduce labor costs	
	T33: RFID technologies increase the operational efficiency of agricultural product supply chains and cut time costs	
Cost	T41: Adopting RFID technologies will increase hardware facility costs	
	T42: Adopting RFID technologies will increase operations and maintenance costs

**Table 5:** Organizational context measurement

Variable	Statement of measurement	References
Upper management support	O21: Upper managers actively respond and pay attention when a project is initiated	Sharma et al. (2008) and Brown and Russel (2007)
	O22: Upper managers support labor resources, finances and materials	
	O23: Upper managers are willing to accept risks when adopting RFID	
	O22: Upper managers inspire employees to apply RFID technologies in the daily work practices	
Trust between enterprises	O31: Enterprises in the agricultural product supply chain have access to a strong mechanism for the distribution of benefits	Yang and Jarvenpaa (2005)
	O31: Enterprises in the agricultural product supply chain maintain strong risk sharing mechanisms	
	O33: Enterprises in the agricultural product supply chain cooperate with one another and promote the adoption of this new form of technology	
Technical knowledge	O41: Enterprises in the agricultural product supply chain have relevant technical knowledge on RFID	Leimeister et al. (2007) and Koh et al. (2011)
	O42: Enterprises in the supply chain have professional staff trained in RFID use	
Employees resistance	O51: Employees resist RFID adoption because they do not trust their own abilities	Bhattacharya et al. (2009)
	O52: Employees worry about losing their jobs as a result of RFID adoption	
	O53: Employees have become accustomed to bar code scanning

**Table 6:** Environmental context measurement

Variable	Statement of measurement	References
Competitive pressure	E11: Competitive pressures force enterprises adopt RFID technologies	Sharma et al. (2008)
	E12: Social features such as cultures and customs affect RFID adoption	
	E13: Partners call for RFID adoption	
Uncertainty	E21: The diversity of consumer demands	Leimeister et al. (2007) and Riggins and Slaughter (2006)
	E22: Consumer demands change frequently	
	E23: Fast-paced technological development	
	E24: Competitors adopt advanced technologies	
Chinese government support	E31: RFID development receives financial support from the Chinese government	Li (2006)
	E32: Relevant policies introduced by the Chinese government boost RFID development	

#### Technological context

See Table 4.

#### Organizational context

See Table 5.

#### Environmental context

See Table 6.

## Data analysis and results

The structural equation modeling (SEM) method was used to test the research model presented in Fig. 1. The two-step approach presented by Anderson and Gerbing (1988) was used. First, the measurement model was estimated through a confirmatory factor analysis (CFA) to test the reliability and validity of the measurement model. The structural model was then analyzed to examine the overall model fit.

### The measurement model

Through survey data analysis, it is easy to obtain the Cronbach’s alpha reliability coefficient of latent variables (see Table 7). As is shown in Table 7, all Cronbach’s alpha values exceeded the 0.70 threshold (Nunnally 1978), demonstrating adequate internal consistency. The Cronbach’s alpha estimates clearly denote reliability.

**Table 7:** Cronbach’s alpha reliability coefficient of latent variables

Categories	Latent variables	Number of items	Cronbach’s alpha
Technological context	Technological complexity	3	0.710
	Technological compatibility	3	0.723
	Perceived effectiveness	3	0.810
	Cost	2	0.922
Organizational context	Upper management support	4	0.789
	Trust between enterprises	3	0.791
	Technical knowledge	2	0.814
	Employee resistance	3	0.865
Environmental context	Competitive pressure	3	0.914
	Uncertainty	4	0.817
	Chinese government support	2	0.866
Adoption	Willing to adopt	2	0.927

The following results were derived from a Chi square test conducted using the AMOS 17.0 software program: CMIN = 583.32, p = 0.03, and at the 0.05 significance level, the null hypothesis is not rejected, denoting that the model fits well. Meanwhile, as is shown in Table 8, the other fitting indexes are GFI = 0.961, NNFI = 0.83, CFI = 0.912, and RSMEA = 0.063. The NNFI values are close to the rational values, and the remaining indicators fall within an acceptable range. The measurement model thus exhibits reasonable model fit to the data.

**Table 8:** Fit indexes of the measurement model

Fit indices	Recommended value^a^	Actual value
Chi square	Lower values are better	583.32
Comparative Fit Index (CFI)	>0.90	0.912
Goodness-of-Fit Index (GFI)	>0.80	0.961
Non-normed Fit Index (NNFI)	>0.90	0.83
Root mean square error of approximation (RMSEA)	<0.1, adequate goodness of fit; <0.05, strong goodness of fit	0.063

^a^Recommended values for concluding “good” model fit to the data (Hair et al. 1998)

### The structural model

The purpose of this study was to develop a stronger understanding of factors that influence RFID adoption in the agricultural product distribution industry and of the degree to which each factor has an effect. To test influencing factors, twelve hypotheses were proposed in “Hypotheses and the research model” section. A path coefficient (regression coefficient among latent variables) significance test similar to the parameter significance test conducted for the regression analysis was then conducted. We used AMOS software to conduct our path analysis. The AMOS software program offers a simple and effective way to determine the critical ratio (CR), which is a *Z* statistic based on the ratio of parameter estimates and their standard deviations. When using the AMOS software for analyses, the *p* value is given at the same time. We thus carried out our test based on the *p* value. A significance level of 0.01 was used in this study. Table 9 presents standard estimates of the path coefficient, the significance level, and the results. As is shown in Table 9, all the hypotheses were supported except for Hypothesis 9 and Hypothesis 11.

**Table 9:** Standard estimates of the path coefficient and the significance level

Hypothesis	Path: from → to	Standard estimate of path coefficient	CR	Significance level	Results
H1	Technological complexity → Adoption	−0.345	−6.74	***	Supported
H2	Technological compatibility → Adoption	0.362	7.621	***	Supported
H3	Perceived effectiveness → Adoption	0.413	7.67	***	Supported
H4	Cost → Adoption	−0.496	−10.347	***	Supported
H5	Organization size → Adoption	0.415	9.342	***	Supported
H6	Upper management support → Adoption	0.375	10.171	***	Supported
H7	Trust between enterprises → Adoption	0.317	8.911	***	Supported
H8	Technical knowledge → Adoption	0.354	7.636	***	Supported
H9	Employees resistance → Adoption	−0.027	−0.07	0.036	Not supported
H10	Competitive pressure → Adoption	0.247	5.068	***	Supported
H11	Uncertainty → Adoption	−0.015	−1.462	0.143	Not supported
H12	Government support → Adoption	0.383	13.079	***	Supported

*** Significance level: p < 0.01

Based on the TOE framework, we studied factors influencing RFID adoption and used the SEM model and AMOS 17.0 software to carry out an empirical analysis of the agricultural product distribution industry in China. We found that the model accurately reflects RFID adoption in the agricultural product supply chain.

**Fig. 2:**
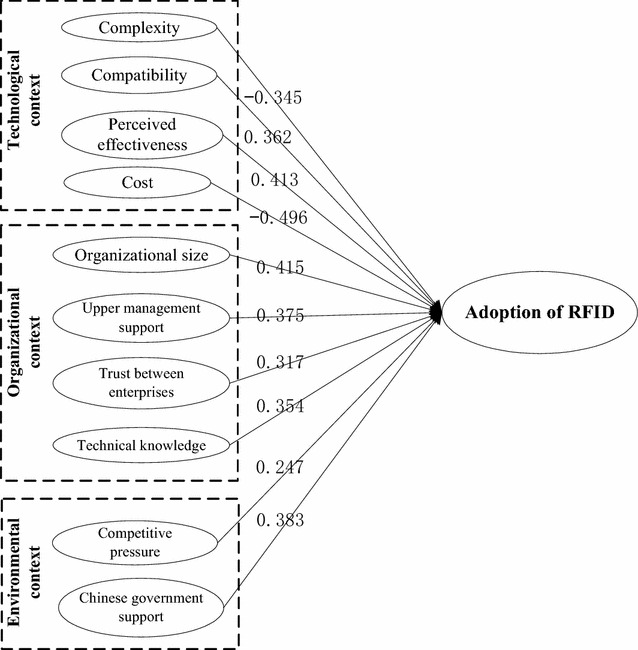
Structural model results

The authors used the AMOS software program to conduct a path analysis. Ten of the twelve hypotheses were supported. The path coefficient results are shown in Fig. 2. It is important to note that the number listed on each arrow denotes the effect of each factor. From our path coefficient significance test, it is clear that all of the factors except for employee resistance and uncertainty are supported by the model. Technological compatibility, perceived effectiveness, organization size, upper management support, trust between enterprises, technical knowledge, competitive pressure levels and government support, which are supported by the model, have significant positive effects on RFID adoption. Among these, organization size has the strongest positive effect, while competitive pressure has the least significant positive effect. Technological complexities and costs have significantly negative effects on RFID adoption. Among them, costs constitute the most significantly negative influencing factor.

## Conclusions

Through our literature review and field research and based on the TOE theoretical framework, we analyzed factors that influence RFID adoption in the Chinese agricultural product distribution industry based on three contexts: the technological, organizational, and environmental contexts. According to the results of our empirical analysis, technological contexts involving technological complexities, technological compatibility, perceived effectiveness, and costs; organizational contexts involving organization sizes, upper management support, trust between enterprises, and technical knowledge; and environmental contexts involving competitive pressures and support from the Chinese government have significant effects on RFID adoption in the Chinese agricultural product distribution industry. Our research findings will help enterprises in the agricultural products supply chain develop a stronger understanding of factors that shape RFID adoption in the agricultural product distribution industry. By making enterprises more aware of how much various factors affect RFID adoption, our findings can help enterprises make more appropriate and rational decisions, thus facilitating RFID adoption in the agricultural product distribution industry.

## Limitations and future research

The following limitations were encountered in this study. The purpose of this study was to develop a stronger understanding of factors that shape RFID adoption in the Chinese agricultural product distribution industry. A small sample was used to gather quantitative and qualitative data. Due to difficulties associated with collecting extensive data, we only studied a small number of samples, meaning that the results of this study require further validation. In future studies, the scope of study samples should be expanded to obtain as much data as possible, making consequent findings more accurate. In addition, numerous other factors functioning outside of and within technological, organizational, and environmental contexts that shape adoption outcomes were not considered in this study.

Although our intention was to develop a balanced perspective of RFID by obtaining views from an equal number of business and IT managers, for many of the organizations studied, the researcher conducting the interviews was directed to IT personnel. Thus, this balance was not achieved. At the time of the study, RFID was still only initially being considered in of many of the Chinese agricultural product distribution enterprises examined. Many business executives therefore still viewed it as a technology-related issue rather than a business imperative.

Our empirical study was carried out in China, and thus the results may not be directly applicable to other countries due to cultural differences. Consequently, related studies should be conducted in different countries. Future studies could use the framework presented here to assess and compare RFID adoption patterns in several countries. In turn, the effects of national environments on RFID adoption can be identified.

## References

[CR1] Abad E, Palacio F, Nuin M (2009). RFID smart tag for traceability and cold chain monitoring of foods: demonstration in anintercontinental fresh fish logistic chain. J Food Eng.

[CR2] Amador C, Emond J-P, Nunes C (2009). Application of RFID technologies in the temperature mapping of the pineapple supply chain. Sens Instrum Food Qual Saf.

[CR4] Anderson JC, Gerbing DW (1988). Structural equation modeling in practice: a review and recommended two-step approach. Psychol Bull.

[CR5] Bernardi P, Demartini C, Gandino F (2007). Agri-food traceability management using a RFID system with privacy protection. Adv Inf Netw Appl.

[CR6] Bhattacharya M, Chu CH, Mullen T (2009) RFID implementation in retail industry: current status, issues, and challenges. In: Decision Science Institute (DSI) conference 2007, Phoenix, AZ, pp 201–203

[CR7] Brown A, Bakhru A (2007). Information systems innovation research and the case of RFID. Int Fed Inf Process.

[CR8] Brown I, Russel J (2007). Radio frequency identification technology: an exploratory study on adoption in the South African retail sector. Int J Inf Manag.

[CR9] Chau PYK, Tam KY (1997). Factors affecting the adoption of open systems: an exploratory study. MIS Q.

[CR10] Fichman RG (1992) Information technology diffusion: a review of empirical research. In: Thirteenth international conference on information systems, Dallas, TX, pp 195–206

[CR11] Geng XF (2005). Application of RFID technique in the supply management. Logist Sci-Tech.

[CR12] Grandon EE, Pearson JM (2004). Electronic commerce adoption: an empirical study of small and medium US businesses. Inf Manag.

[CR13] Gras D (2006) RFID based monitoring of the cold chain. In: 2nd International workshop cold chain management

[CR14] Hair JF, Anderson RE, Tatham RL, Black WC (1998). Multivariate data analysis.

[CR15] Jedermanna R, Ruiz-Garciab L, Lang W (2009). Spatial temperature profiling by semi-passive RFID loggers for perishable food transportation. Comput Electron Agric.

[CR16] Koh CE, Kim HJ, Kim EY (2011). The impact of RFID in retail industry: issues and critical success factors. J Shopp Center Res.

[CR17] Kuan KK, Chau PY (2001). A perception-based model for EDI adoption in small businesses using a technology–organization–environment framework. Inf Manag.

[CR18] Leimeister JM, Knebel U, Krcmar H (2007). RFID as enabler for the boundless real-time organization: empirical insights from Germany. Int J Netw Virtual Organ.

[CR19] Li YW (2006) A factors comparative research on organizational adoption behavior between pre-adoption and post-adoption. PhD thesis, Tongji University

[CR20] Li WC (2011) Study on process model of RFID adoption and its application issues for automotive manufacturing enterprises. PhD thesis, Chongqing University

[CR21] Liu D, Tang J (2010). Construction of tracing and monitoring system of dairy products. J Northeast Agric Univ.

[CR22] Nunnally JC (1978). Psychometric theory.

[CR23] Premkumar G, Ramamurthy K (1995). The role of inter-organizational and organizational factors on the decision mode for adoption of inter-organizational systems. Decis Sci.

[CR24] Regattieri A, Gamberi M, Manzini R (2007). Traceability of food products: general framework and experimental evidence. J Food Eng.

[CR25] Riggins FJ, Slaughter KT (2006) The role of collective mental models in IOS adoption: opening the black box of rationality in RFID deployment. In: Proceedings of the 39th Hawaii international conference on system sciences (HICSS), Hawaii, HI, pp 183–184

[CR26] Rogers EM (1983). Diffusion of innovations.

[CR27] Sahin E, Dallery Y, Gershwin S (2002) Performance evaluation of a traceability system: an application to the radio frequency identification technology. In: Proceedings of the 2002 IEEE international conference on systems, man and cybernetics, vol 3 pp 210–218

[CR28] Seymour L, Lambert PE, Willuweit L (2010) RFID adoption into the container supply chain: proposing a framework. In: Proceedings of the 6th annual ISOnEworld conference, Las Vegas. IEEE, pp 431–435

[CR29] Sharma A, Thomas D, Konsynski B (2008) Strategic and institutional perspectives in the evaluation, adoption and early integration of radio frequency identification (RFID): an empirical investigation of current and potential adopters. In: Proceedings of the 41st Hawaii international conference on system sciences (HICSS), Hawaii. IEEE, pp 407–407

[CR30] Tornatzky LG, Fleischer M (1990). The process of technological innovation.

[CR31] Tornatzky LG, Klein K (1982). Innovation characteristics and innovation adoption-implementation: a meta-analysis of findings. IEEE Trans Eng Manag.

[CR32] Xie J, Hu Y, Liu C (2007). WSNs-and-RFID-based safety monitoring platform for agricultural products. Chin Agric Mech.

[CR33] Yang G, Jarvenpaa SL (2005) Trust and radio frequency identification (RFID) adoption within an alliance. In: Proceedings of the 38th Hawaii international conference on system science, pp 1–10

[CR34] Zhang DQ (2011). The application and problems of RFID in the construction of agricultural products quality safety system in China. Acad Exch.

[CR35] Zhang DH, Kang SY (2008). Influence factors and countermeasure analysis on technology adoption for logistics information grid. J Inf.

